# Basic life support knowledge in Germany and the influences of demographic factors

**DOI:** 10.1371/journal.pone.0237751

**Published:** 2020-08-20

**Authors:** Jennifer Lynn Schiefer, Hannelore Schuller, Paul Christian Fuchs, Mahsa Bagheri, Daniel Grigutsch, Matthias Klein, Alexandra Schulz

**Affiliations:** 1 Clinic of Plastic, Reconstructive, Hand and Burn Surgery, Hospital Cologne Merheim, University of Witten-Herdecke, Witten, Germany; 2 Clinic of Anesthesiology at the University Hospital Bonn, Bonn, Germany; 3 Emergency Department and Department of Neurology Hospital of the Ludwig-Maximilians-University (LMU) Munich, Munich, Germany; University of Alberta, CANADA

## Abstract

**Background:**

In the developed world, cardiovascular diseases still contribute to mortality and morbidity, leading to significantly increased deaths in recent years. Thus, it is necessary for a layperson to provide the best possible basic life support (BLS) until professional help is available. Since information on current BLS knowledge in Germany is not available, but necessary to be able to make targeted improvements in BLS education, we conducted this study.

**Methods:**

A cohort survey using convenience sampling (non-probability) method was conducted with questions found in emergency medicine education. People coming to the emergency room of two big university hospitals located in the South (Munich) and western part (Cologne) of Germany were asked to participate in the survey between 2016 and 2017. Primary outcome measures were the proportion of correct answers for each emergency scenario in relationship to age, region, profession and first-aid training.

**Results:**

Altogether 1003 people (504 from Cologne; 499 from Munich) took part in the questionnaire. 54.7% were female and 45.3% were male aging from 19 to 52 with a mean of 37.2 years. Although over 90% had taken part in first aid training, many people were lacking first aid knowledge, with less than 10% choosing the correct frequency for chest compression. Hereby demographic factors had a significant influence (p<0.05) in the given answers (Friedmann-and-Wilcoxon Test).

**Conclusion:**

Overall, results of our survey indicate a clear lack of BLS knowledge. With this information, targeted measures for improving BLS knowledge should be conducted. Additionally, further studies on the feasibility and efficiency of teaching methods are needed

## Background

In the developed world, cardiovascular diseases contribute significantly to mortality and morbidity. The number of related deaths have risen significantly from approximately 12.3 million in 1990 to 17.6 million in 2016 (+ 43.1%) [1, 2]. Thus, there is an urgent need of knowledge on how to deal correctly with victims as a bystander.

BLS procedures encompass a number of emergency techniques to sustain patient’s life following a cardiac arrest until advanced medical care is provided. BLS, including cardiopulmonary resuscitation (CPR) and usage of automated external defibrillators (AED), combines different skills such as chest compression (CC) and mouth-to-mouth breathing in order to recover blood circulation to patient’s vital organs and to the brain. Importance of CPR stems from the fact that a laypersons application of CPR and AED are deemed as fundamental in American Heart Association’s (AHA) Chain of Survival [3]. A patient’s survival relies on a quick and correct first aid treatment, i.e. it depends on an adequate knowledge and suitable awareness of basis techniques of the helpers [4, 5]. Thus, official institutions provide guidelines with a set of systematized standard procedures. In Europe, the European Resuscitation Council (ERC) (www.erc.edu) guidelines of BLS activities came into effect in 2010 and its intended to update every five years [6, 7]. However, promulgation and effects of these recommendations are widely unknown.

Previous literature reports from European researchers indicate a pervasive consensus that there is an unmet need to strengthen BLS knowledge among the general society. Despite a large number of educational initiatives, there is no clear evidence of the most effective method [8]. A Norwegian study [4] on first aid in cases of out-of-hospital cardiac arrest (OHCA) has shown that a large share of the general population in Norway experienced first aid trainings based on compulsory national school curriculum but theoretical first aid knowledge on OHCA or trauma turned out to be worse than expected. A Polish survey [9] among participants of rock music festivals suggested a permanent need to enhance both BSL knowledge and skills of the population under scrutiny as well as of the general society. It was concluded that there is a necessity for regular teaching of official first aid rules in accordance with current provisions from institutions [8].

In Germany, little information about BLS knowledge among the general population is available. Analysis of registered data (Deutsches Reanimationsregister) of period 2004–2012 has shown that in only 16.1 percent of the reported OHCA cases first aid was provided by bystanders [10], a value that proved to be at a similar level with Poland (27%) and Romania (6%) but is very low compared to other European countries such as Sweden or Netherlands (> 60%) [10]. The low value may be indicative for the low level of BSL/CPR knowledge among German population. Not surprisingly, the overall survival rate after OHCA incidents has not significantly increased over the last years despite medical strides, probably a consequence of inadequate registration of comparable data [11, 12], which is presumably abolished in part by introduction of ERC guidelines. Hence, we think it is highly desirable to understand and establish whether BLS/CPR knowledge from official recommendations is being spread among the German lay population. In order to gain insight to the level of BLS knowledge held by the common society, we decided to conduct a survey among visitors of emergency departments of two German hospitals reflecting typical features of an urban city as well as of its municipal and rural areas around [11]. Therefore, our aim was to investigate BLS knowledge in Germany, in order to provide a standard baseline for coherent future recommendations in BLS training,

## Methods

A cohort survey using convenience sampling (non-probability) method was conducted using 16 standard questions found in various German emergency medicine education literature (Table 1). The questions and scenarios have been taken from questionnaires typically used for first-aid-training and found in literature [13, 14]. Further validation prior to this survey has not been performed. The study was approved by the ethical committee of the University of Witten/Herdecke number:110/2018. The ethics committee waived the need for consent.

**Table 1:** Answers given to the first aid questions and scenarios.

First aid questions and scenarios		Cologne (n = 504)		Munich (n = 499)	
1	**Did you have to admit first aid previously?**	n	%	n	%
	Yes	172	34,1	159	31,9
	No	332	65,9	340	68,1
2	**If yes, how was your reaction to this situation?**				
	A. I knew what to do but others were faster	26	5,2	17	3,4
	B. I knew what to do and helped as well as possible	121	24,0	107	21,4
	C. I was unsure, but tried to help as well as I could	23	4,6	28	5,6
	D. I did not know what to do and therefore did not help	2	,4	0	0,0
	E. No answer	332	65,9	347	69,5
3	**What is the European emergency number**				
	A.112	445	88,3	390	78,2
	B. 19222	16	3,2	46	9,2
	C. 110	38	7,5	59	11,8
	D. 116117	2	,4	3	,6
	E. No answer	3	0,6	1	,2
4	**What has to be done if a person does not react anymore, but is still breathing normally?**				
	A. Bring into abdominal position	6	1,2	13	2,6
	B. Bring into side position	469	93,1	451	90,4
	C. Start with reanimation	11	2,2	13	2,6
	D. Do not move- just leave the person lying there	14	2,8	21	4,2
	E. Don't know	4	,8	1	,2
5	How often are you supposed to press per minute during cardiac massage of an adult				
	A. 10 times	119	23,6	172	34,5
	B. 30 times	240	47,6	213	42,7
	C. 60 times	104	20,6	86	17,2
	D. 100 times	30	6,0	24	4,8
	E. Don't know	2	,4	4	,8
	No answer	9	1,8		
6	**Where do you have to press during cardiac massage?**				
	A. On the heart	52	10,3	51	10,2
	B. Between the nipples	317	62,9	298	59,7
	C. In the middle of the thorax	68	13,5	96	19,2
	D. On the upper third of the sternum	57	11,3	50	10,0
	E. Don't know	10	2,0	4	,8
7	**A 49-year-old employee becomes unconscious in his office; normal breathing cannot be detected. What would you do?**				
	A. Emergency call, wait for the specialists	30	6,0	50	10,0
	B. Emergency call, cardiac massage, better no mouth-to-mouth resuscitation	31	6,2	29	5,8
	C. Emergency call, cardiac massage and mouth-to-mouth resuscitation 10:2	193	38,3	211	42,3
	D. Emergency call, cardiac massage and mouth-to-mouth resuscitation 30:2	224	44,4	187	37,5
	E. Don't know	25	5,0	22	4,4
	No answer	1	0,2		
8	**His colleague administers first aid through cardiopulmonary resuscitation. After a short time, a further colleague brings an automated external defibrillator (AED). What would you do?**				
	A. I do not know how the AED functions- better won't use it.	35	6,9	46	9,2
	B. I have never heard anything about a AED	25	5,0	20	4,0
	C. Switch AED on and follow instructions	379	75,2	365	73,1
	D. I have heard something about an AED, but I do not know for what you use it.	13	2,6	13	2,6
	E. Don't know	51	10,1	55	11,0
	No answer	1	0,2		
9	**If a person has a heart attack, where would he or she feel pain most likely?**				
	A. In the breast	466	92,5	466	93,4
	B. In the lower stomach region	5	1,0	7	1,4
	C. In the legs	3	,6	7	1,4
	D. Don't know	29	5,8	19	3,8
	No answer	1	0,2		
10	**A 45 year old man has an asthma attack, What would you do?**				
	A. Help the person to sit in a comfortable and upright position and take his medication	304	60,3	145	29,1
	B. Help the person to sit in a comfortable and upright position and breathe in a paper bag	91	18,1	70	14,0
	C. Tell the person to do some stretching exercises and walk around the block	5	1,0	30	6,0
	D. Let the person breathe in and out deeply and get a glass of water to drink	65	12,9	153	30,7
	E. Don't know	38	7,5	100	20,0
	No answer	1	0,2	1	,2
11	**An 80-year-old woman chocked on something. What would you do?**				
	A. Encourage her to breathe through her nose	52	10,3	57	11,4
	B. Help wash the foreign object down with water	100	19,8	91	18,2
	C. Hit the woman hard on the back between the shoulder blades	309	61,3	318	63,7
	D. Don't know	42	8,3	33	6,6
	No answer	1	0,2		
12	**A 25-year-old man has a seizure. What would you do to help?**				
	A. Stuff something into his mouth	53	10,5	71	14,2
	B. Hold him tight	88	17,5	81	16,2
	C. Make sure he doesn't hurt himself	322	63,9	296	59,3
	D. Don't know	40	7,9	48	9,6
	No answer	1	0,2	3	0,6
13	**A 30-year-old woman is unconscious, but still breathing. What can you do to maintain the airways**				
	A. Roll the woman on the side and tilt her head back.	380	75,4	413	82,8
	B. Place the woman on her belly and tilt her head back	8	1,6	9	1,8
	C. Make sure nothing blocks the nose	84	16,7	46	9,2
	D. Don't know	31	6,2	31	6,2
	No answer	1	0,2		

The study was planned to include 1000 people. With this sample size incidence rates respective 95% confidence intervals would have a precision of +/- 2–3% (2% in case of a 10% prevalence, and 3% in case of a 50% prevalence).

From January 2016 until August 2017 all patients, visitors and medical personnel over 18 years of age in the emergency room of the University hospitals Munich (South of Germany) and Cologne-Merheim in the Western part of Germany (University of Witten/Herdecke) were asked to take part in the survey on different randomly picked days (between 7:00 and 19:00) as these regions reflect rural structure through the outskirts of Munich and urban structure though the people living in the cities Cologne and Munich. Additionally people coming to the emergency room often have different ages. That way different from online studies, that might exclude older people and people without the necessary technology, every age, profession and income group was able to take part in our survey, reflecting general population characteristics. Demographic data collected, included the age, gender and profession (Table 2).

**Table 2:** Demographic information of the participants.

Demographic information of participants				
		NRW	Bavaria	total
number		504	499	1003
male (n)		238 (47.2%)	216 (43.3%)	454 (45.3%)
female (n)		266 (52.8%)	283 (56.7%)	549 (54.7%)
age (n)	< 20 years	31 (6.2%)	31 (6.2%)	
	21-25yeara	51 (10.1%)	59 (11.8%)	
	26–30 years	66 (13.1%)	67 (13.4%)	
	31–40 years	86(17.1%)	112 (22.4%)	
	41–50 years	107 (21.2%)	118 (23.4%)	
	> 50 years	163 (32.3%)	112 (22.4%)	
education (n)	middle school	177	160	337
	high school	83	96	179
	apprenticeship	179	132	311
	graduate and undergraduate studies	63	111	174
	no answer	2	0	2
first aid training	yes			331 (33.0%)
	no			672 (67.0%)

Exclusion criteria were age under 18 and the lack of understanding the survey questions. Primary outcome measures of the study were the proportion of correct answers for each emergency scenario in relationship to region, age, profession, and first-aid training.

Since 1999 a basic life support training involving eight 45-minute sessions and since 2015 a first-aid training involving nine 45-minute sessions including is obligatory for receiving a driver’s license in Germany (“Fahrerlaubnisverordnung” §19). Additionally, since 2015 every person having a driver’s license is supposed to update first aid knowledge every 2 years. Though this is not verified [15]. No efforts were undertaken to address potential sources of bias.

### Statistical analyses

Microsoft Excel (2013, Microsoft, USA) was used to manage data and design the charts. Prior to the analysis, data were checked for completeness and plausibility. Statistical analysis was performed with SPSS (IBM, USA) Version 21. The data was collected prospectively. Statistical significance was accepted at p-values < 0.05. The Friedman and Wilcoxon Test were performed to identify statistical significant differences between the subgroups.

## Results

**Fig 1:**
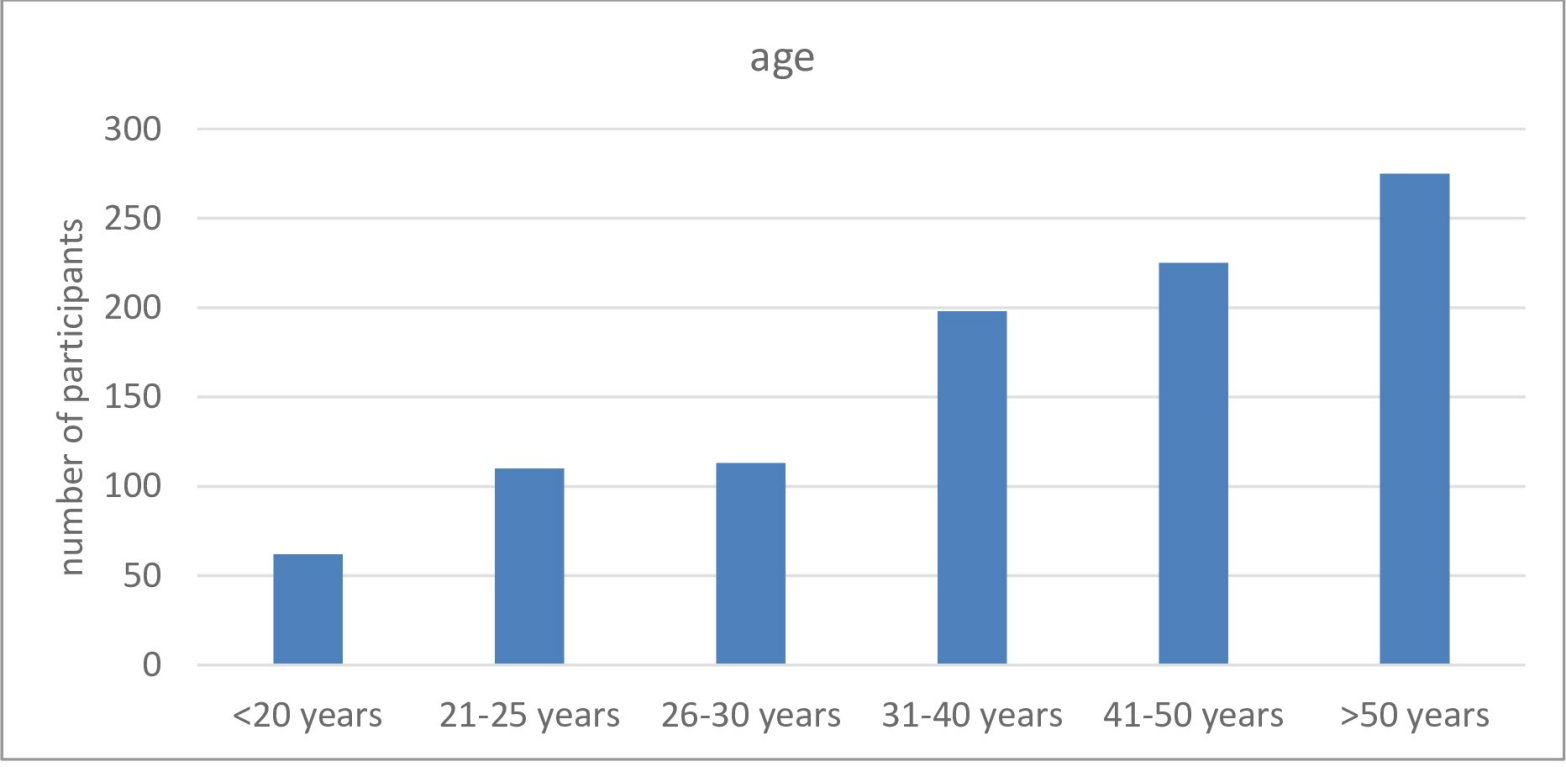
Overview of the age distribution among the questioned people.

Altogether 1003 people took part in the questionnaire. More than half of the people came to the emergency room as a patient. In total, 504 participants were from Cologne (North-Rhein-Westphalia) and 499 from Munich (Bavaria) with 54.7% females and 45.3% males. Their age ranged from 19 to 52 with a mean of 37.2 years (Fig 1). Concerning their education, approximately 17.9% graduated with the so-called German “Abitur”, 31% had a specific training (“Ausbildung”) and 17.4% went to university. The largest group taking part at the survey, were medical personnel (9.4%), followed by 7.1% teachers.

### First aid training

Almost 90% of all participants stated, that they had taken part in first aid training. Hereby almost 30% (29.4% Cologne; 29.9% Munich) had taken part in the training within the last 5 years. More than a third of the participants (38.9%) stated that their training was more than 10 years ago. Approximately 33% of all questioned people and 58.5% of the medical personnel stated, that they had helped in an emergency situation. Hereby altogether 83.7% knew what to do in that situation.

The European emergency number was pointed out correctly significantly more often in Cologne (88.3%) compared to Munich (78.2%; p<0.001).

Over 90% of all questioned people stated correctly, that they would place a person, who does not respond but breathes normally on his side. Only 25 people would start reanimating this person, in contrast to 35 people who would not move and touch the person.

### Reanimation

29% of all people suggested to perform heart massage 10 times a minute, whereas only 5.3% of all questioned people and 9.6% of all questioned medical personnel wanted to perform heart massage with a rate of 100 per minute, which would be correct. Overall in Cologne significantly more people (6%) would perform chest compression with the correct rate of 100 times per minute compared to only 4.8% of all participants in Munich (p = 0.006).

It was found to be significant more likely, that participants answered question 6 correctly, if they had completed a first aid training previously (p<0.001) or were health professionals (p<0.001). Hereby approximately 60% would correctly press between the nipples, and 10.2% on the heart. A significant difference between Cologne and Munich could not be detected (Table 2).

### Cardiac arrest

After finding an unconscious person who was no longer breathing, 6% in Cologne and 10% in Munich would call the emergency hotline and wait until they come. All other people would correctly start immediately with the reanimation after calling the emergency hotline.

If confronted with an external defibrillator over 75% of all questioned people would switch it on and correctly wait for further instructions. 4.5% of all questioned people had no clue what it is supposed to be used for and 10.6% were not sure how to use it. Significant differences between Cologne and Munich were not registered.

### Case scenarios

Over 60% of all questioned people and 76.2% of first aiders would accurately let a person with an asthma attack sit in an upright position and help him or her take the necessary medication. Unfortunately, 17.4% would let the person with an asthma-attack breathe in a paper bag. Solely 1.4% would tell the person to stretch and go for a walk.

In the case of an old lady who choked on something, over 60% of all questioned people and 87.6% of first aiders would correctly hit her between the shoulder blades, 19% would get a glass of water to drink and approximately 10% would tell the lady to breathe through her nose.

Further, 61.6% would correctly attempt to ensure a man with a seizure does not hurt himself (p = 0.03). Though 12.3% would try to stuff something into his mouth.

Among all people asked, 79.1% would roll an unconscious woman that is breathing on to her side, with significant more people (82.8%) choosing the right action in Munich, than in Cologne (75.4%; p = 0.006).

**Fig 2:**
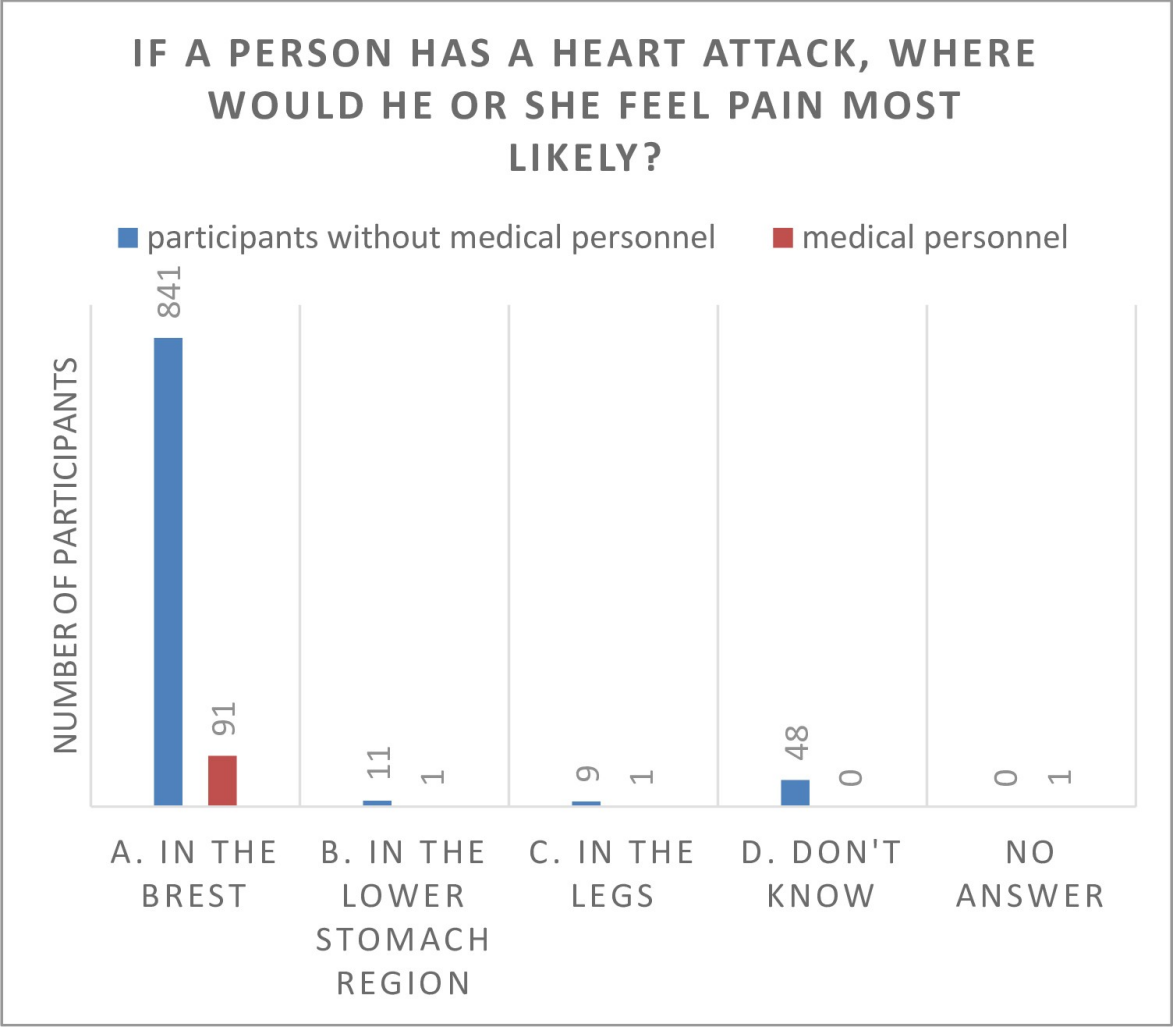
Answers given to question number 8 from medical personnel and other professions in comparison.

**Fig 3:**
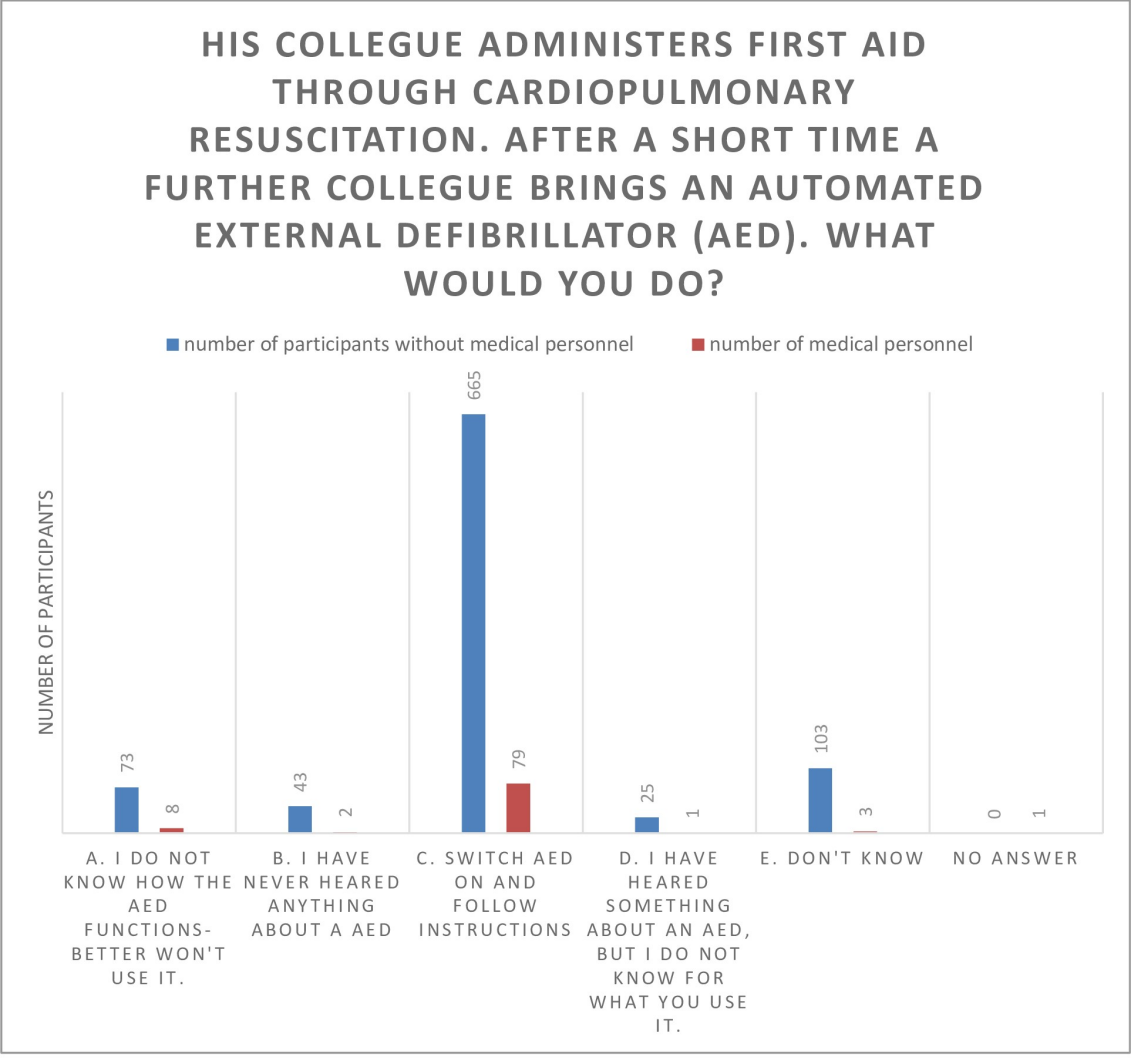
Answers given to question number 9 from medical personnel and other professions in comparison.

There was no statistically significant influence of age in the number of correct answers in the survey. Though, we found significant different answers for health care professionals and participants from other professions in the following two fields (Figs 2 and 3):

#### Reanimation

Compared to only 78% of the participants of other professions, significantly more health care professionals (89.4%) would turn an unconscious patient in a stable lateral position (p = 0.005).

#### Cardiac arrest

When it comes to the use of an automated external defibrillator (AED), significant more health care professionals (84% versus 73.20%) would dare to switch on the device and follow the instructions. In contrast to the health care professionals asked, more people from other professions have never heard about the AED (4.7% versus 2.1%) or they had no idea how to handle the question (11.3% versus 3.2%; p = 0.013). Compared to non-health care professionals, people in health care taking place in our survey knew significantly more often, where the pain of an heart attack might be located (96.8% versus 92.5%; p = 0.055).

## Discussion

The lack of consistent baseline information and teaching standards have made it difficult to find a targeted approach to train citizens in BLS/CPR. Thus, we aimed to evaluate the current knowledge of BLS among German lay populace. Overall, results of our survey were mixed and indicate a clear lack of BLS/CPR knowledge.

We found highest proportions of correct answers in application of positioning of the patient (unconscious patient but with breathing) and identification of heart attacks (> 90% or > 75% correct answers, respectively). On the other side, in some cases **a clear lack of knowledge** has been found concerning CC and/or mouth-to-mouth resuscitation (< 10% correct answers). In all other scenarios, a moderate share of correct answers has been found.

The picture is in line with the body of literature from other countries. An US-American survey [3] among patients in an urban hospital reflects socioeconomic disparities with respect to BLS techniques in addition to a general lack of CRP training and low levels of confidence in performing resuscitation as well as a restricted ability to use AEDs correctly [3]. In an older Polish survey [16], it was found that 75 percent of respondents were trained on CPR, but most of them declared their CPR ability to be inadequate. Another study from Greece addressed the CPR knowledge gap by evaluating the prevalence of CPR training among the Greek community [17]. Here, only 6.5 percent of respondents had attended a CPR course during the last 12 months while knowledge turned out to be poor, indicating a low prevalence of extensive and well-organized CPR trainings. Two Turkish studies [18, 19] have shown that BLS/CPR trainings are not routine outside the health care sector of Turkey, resulting in a poor to moderate knowledge and participation of BLS/CPR courses among general society. In Sweden, a nationwide survey was conducted in the early 2000s to find out whether knowledge on CPR has been disseminated across populace [20]. Results showed that up to 45 percent of respondents had participated in CPR training, without dealing with the knowledge in detail.

First aid education in Germany is often administered during the attendance of driver’s license training or in occupational settings. Thus, we can confirm a high share (90%) of the populace who attended BLS trainings, but this seems to be ineffective with respect to OHCA [21]. The high share of first aid course attendees is similar to that in the Norwegian survey provided by Bakke et al. (2017), where first aid courses are part of the national school curriculum, resulting in a similar high proportion of formally trained responders. In Germany, such BLS education programs seems to be in the ascendant [10, 22, 23]. The share of first aid trained people in Germany is considerable higher compared to reports from Sweden and non-European regions (USA, Australia/New Zealand), where the share ranged from 45–79 percent [4]. On the other hand, the proportion of those respondents who attend a first aid course within the last five years in our study were only 29–30 percent compared to 54 percent in Norway, although the random sample of this survey can be regarded as less representative than ours due to several potential biases of the interviewees [4]. It should be noted that comparison with literature reports are hampered by the fact that most studies are focussed solely on CPR trainings [4], or using the terms BLS and CPR in an interchangeable way, or are dealing often with very specific groups of respondents.

The questionnaire used, is broadened by other specific questions on different case studies as resuscitation and asthma as well as more common knowledge such as the emergency number. With respect to demographic reasons, knowledge is tentatively lower in regions with low income and lower educational level [3].

### Implications

Following our results, we advocate for the improvement of targeted BLS training in German schools [24]. Schools seem to be an ideal place for BLS/CPR trainings as the Swedish study has shown that foreign born and unemployed people are hardly reached by alternative ways [20]. In Germany, isolated efforts have been made so far to teach CPR to schoolchildren nationwide in order to train the population as a whole [23]. Simpler strategies to reach larger segments of laypersons in an efficient manner did not increase BLS skills in many communities [24]. Anyway, it has been argued that training the populace as a whole would require tremendous effort [20]. Thus, sophisticated CPR-education programs in schools may be a highly promising approach to impart practical skills, to increase theoretical knowledge and to strengthen self-confidence to perform CPR correctly in an integrated way [23]. Certain levels of knowledge and self-confidence are assumed to spread effectively within group members [23]. However, these pilot programs have to be carefully evaluated because relevant factors for designing far-reaching community CPR training programs are not well defined. In this context, smartphone applications might also be helpful in future CPR trainings [25–28].

Furthermore, the term BLS is not uniformly used in literature. It is often confused with first aid, which can encompass aspects beyond resuscitation, i.e. treatment of burns and similar injuries, basic or advanced life support, or even a non-resuscitative kind of support [4]. Thus, more standardization and transparency to prevent confusion should be an integral part of new CPR education programs. Further, establishing networks to design solid studies to test the effectiveness of training methods are needed in the long term. Optimizing educational strategies and identifying why bystanders fail to respond are major knowledge gaps that may affect public health [8, 29]. Especially, as far as recent studies suggest that there is a positive association between increased number of trained bystanders in BLS and the survival rate in out-of-hospital cardiac arrest [30].

### Limitations

1. It has been argued repeatedly that **theoretical knowledge does not necessarily reflect practical first aid skills** [4, 17]. Indeed, correct BLS treatments may be hampered by other reasons than pure knowledge gaps, for instance reservations related to fear of infections [4] or fear of further harming the victim [18]. In a Turkish study, participants with previous emergency experience or BLS training answered significantly more of the theoretical questions correctly, but did not performed significantly better in the practical questions compared to laypersons [18]. One reason for this could be, that certain aspects are not questioned in-depth such as the correct AED usage [3]. The retention of CPR skills has been described as poor among laypersons and even health professionals, in particular when they apply them unregularly [20] or when too much time has passed since last training [17]. In short, bearing the contradiction of a high prevalence of first aid courses and poor BLS application skills in mind, it could be that these new education programs become ineffective as well. Not surprisingly, in the USA a very simple CPR teaching format for laypersons has been developed recently to promote knowledge and willingness to perform CPR in an optimized way [24]. In addition, adolescents and young adults has been regarded as a community less likely to be present when BLS/CPR skills are needed [17]. Recent studies have shown, that instructor-led teaching methods, with hands -on practice supported by real-time feedback show an apparent advantage [8].
2. Our survey is based on a convenience sample, because it did not include all possible hospital visits and only took place between 7:00 am and 7:00 pm [3].
3. A further limitation could be the multiple-choice format of the questionnaire we have chosen, which may lead certain answer patterns due to random guessing [17].
4. Additionally, the exact date, length and content of each individual training is unclear.

## Conclusion

In conclusion, we conducted the first comprehensive survey among German populace to elucidate BLS knowledge linked to demographic factors. Based on a sample of more than 1.000 participants, results show an unregular pattern with only poor to moderate answers. Although this is in line with other European studies, we conclude that official ERC guidelines haven’t been adopted by the society so far and/or that conventional first aid education doesn’t fit a realistic need of BLS practices. Further studies are needed to develop effective teaching methods and implement broadly validated evaluation criteria.

## Supporting information

S1 File Original German questionnaire. (DOCX)
