# Supplementary material for: Basic life support knowledge in Germany and the influences of demographic factors
Source: PLoS One. 2020 Aug 20;15(8):e0237751. doi: 10.1371/journal.pone.0237751 (PMC7446818; doi:10.1371/journal.pone.0237751)
Supplement: S1 File — (DOCX) [file pone.0237751.s001.docx]

**Fragebogen zur Ersten Hilfe**

**A. Allgemein**

**1. Mussten Sie schon einmal Erste Hilfe leisten (beruflich oder privat)?**

- Ja
- Nein

**Wenn ja - wie war Ihre Reaktion auf diese Situation?**

1. Ich wusste was ich tun musste, aber andere hatten bereits geholfen.
2. Ich wusste was ich tun musste und habe geholfen, so gut ich konnte.
3. Ich war mir unsicher, habe aber trotzdem nach besten Wissen und Gewissen geholfen.
4. Ich wusste nicht was ich zu tun habe und habe auch nicht geholfen.

**2. Wie lautet die europaweit einheitliche Notrufnummer?**

1. 112
2. 19222
3. 110
4. 116117

**3. Welche Maßnahme muss bei einer Person durchgeführt werden, die nicht mehr ansprechbar ist, aber noch (normal) atmet?**

1. In die Bauchlage bringen
2. In die Seitenlage bringen
3. Wiederbelebung durchführen
4. Nicht bewegen – einfach liegen lassen

**4. Wie oft sollte man bei einer Herzdruckmassage bei einem Erwachsenen pro Minute drücken?**

1. 10x drücken
2. 30x drücken
3. 60x drücken
4. 100x drücken

**5. Wo muss man bei einer Herzdruckmassage genau drücken?**

1. auf das Herz
2. zwischen die Brustwarzen
3. in die Mitte des Brustkorbes
4. am oberen Brustbeindrittel

**B Fallbeispiele:**

*Hinweis: In allen folgenden Fällen geht es um Erste-Hilfe Maßnahmen innerhalb der ersten 10-15 Minuten nach der Verletzung.*

**Asthma/Kreislaufkollaps/Krampf**

**1. Ein 45-jähriger Mann hat einen Asthmaanfall. Was machen Sie?**

1. Der Person helfen sich in eine bequeme und aufrechte Position zu setzen und die Medikamente zu nehmen
2. Der Person helfen sich in eine bequeme und aufrechte Position zu setzen und in eine Papiertüte zu atmen
3. Die Person anweisen Streckübungen zu machen und eine Runde um den Block zu laufen
4. Die Person tief ein- und ausatmen lassen und ein Glas Wasser zum Trinken holen
5. Keine Ahnung

**2. Eine 80-jährige Dame hat sich verschluckt. Was machen Sie?**

1. Die Dame ermuntern durch die Nase zu atmen
2. Beim Trinken von Wasser helfen um den Fremdkörper herunter zu spülen
3. Die Dame fest auf den Rücken zwischen die Schulterblätter schlagen
4. Keine Ahnung

**3. Ein 25-jähriger Mann hat einen Krampfanfall. Was würden Sie tun um zu helfen?**

1. Etwas in seinen Mund stopfen
2. Ihn festhalten/fixieren
3. Den Mann vor Verletzungen bewahren
4. Keine Ahnung

**4. Eine 30-jährige Frau ist bewusstlos, atmet aber noch. Was kann man tun um die Atemwege frei zu halten?**

1. Die Frau auf die Seite rollen und den Kopf in den Nacken legen
2. Die Frau auf den Bauch legen und den Kopf in den Nacken legen
3. Sicher stellen, dass nichts die Nase blockiert
4. Keine Ahnung

**Herzstillstand**

**5. In seinem Büro bricht ein 49-jähriger Mitarbeiter bewusstlos zusammen; eine normale Atmung ist nicht feststellbar. Was würden Sie machen?**

1. Notruf, auf die Spezialisten warten
2. Notruf, Herzdruckmassage lieber keine Atemspende
3. Notruf, Herzdruckmassage und Atemspende im Wechsel 10:2
4. Notruf, Herzdruckmassage und Atemspende im Wechsel 30:2
5. Keine Ahnung

6. **Seine Kollegin leistet Erste Hilfe durch eine Herz- Lungen-Wiederbelebung. Nach kurzer Zeit hat ein weiterer Mitarbeiter einen automatisierten externen Defibrillator (AED) geholt. Was würden Sie machen?**

1. Ich weiß nicht wie der AED funktioniert - lieber nicht einsetzen
2. Ich habe noch nie etwas von einem AED gehört
3. AED anschalten und Anweisungen folgen
4. AED habe ich schon mal gehört, weiß aber nicht wofür man ihn einsetzt
5. Keine Ahnung

**7. Wenn eine Person einen Herzinfarkt hat, wo würde diese am ehesten Schmerzen spüren?**

1. In der Brust
2. In der unteren Bauchregion
3. In den Beinen
4. Keine Ahnung
